# Supplementary material for: Dispersal dynamics of SARS-CoV-2 lineages during the first epidemic wave in New York City
Source: PLoS Pathog. 2021 May 20;17(5):e1009571. doi: 10.1371/journal.ppat.1009571 (PMC8136714; doi:10.1371/journal.ppat.1009571)
Supplement: S1 Fig — We delineated those clades by performing a discrete phylogeographic reconstruction along a time-scaled phylogenetic tree while only considering two potential ancestral locations: “New York State” and “other location”. We identified a minimum number of 116 lineage introductions (95% HPD interval = [107–127]), which showcases the relative importance of external introductions considering the number of sequences sampled in our labs during the first wave in New York State (n = 828). On the phylogenetic tree, lineages circulating in New York State are highlighted in purple, and larger purple nodes correspond to the most ancestral node of each clade. (*) refers to the most recent common ancestor inferred for the major New York State clade on which we focused in the second part of this study dedicated to integrated continuous and discrete phylogeographic inference based on downsampled subsets of SARS-CoV-2 genomic sequences. In the lower-left corner, we report the distribution of the sizes of New York State clades. (**) For ease of visualization, the major state clade comprising 596 genomic sequences is not shown in the histogram. (PDF) [file ppat.1009571.s001.pdf]

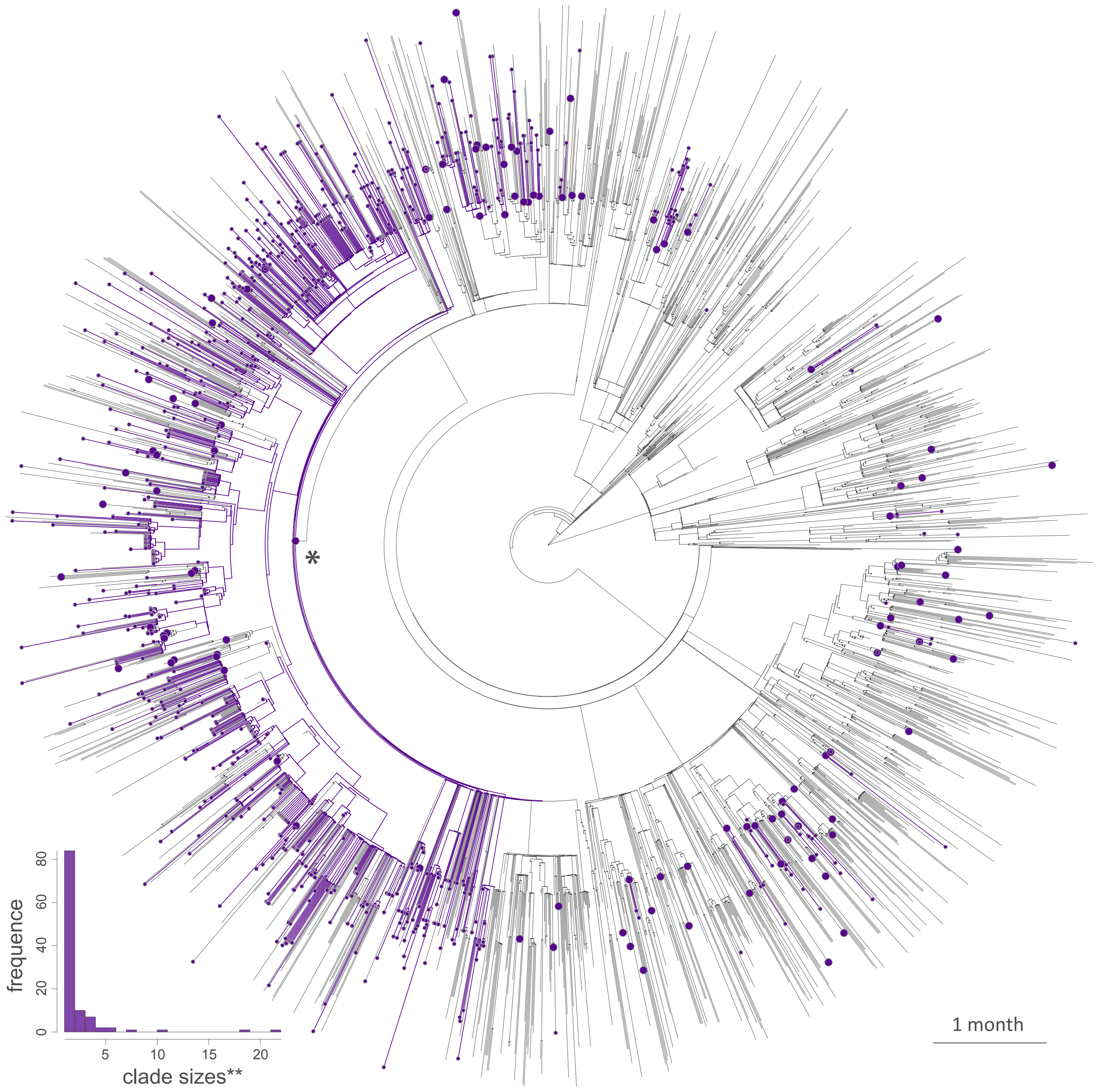

Figure S1. Time-scaled phylogenetic tree in which we identified SARS-CoV-2 phylogenetic clades introduced in New York State during the first epidemic wave. We delineated those clades by performing a discrete phylogeographic reconstruction along a time-scaled phylogenetic tree while only considering two potential ancestral locations: ?New York State? and ?other location?. We identified a minimum number of 116 lineage introductions (95% HPD interval = [107-127]), which showcases the relative importance of external introductions considering the number of sequences sampled in our labs during the first wave in New York State (n = 828). On the phylogenetic tree, lineages circulating in New York State are highlighted in purple, and larger purple nodes correspond to the most ancestral node of each clade. (*) refers to the most recent common ancestor inferred for the major New York State clade on which we focused in the second part of this study dedicated to integrated continuous and discrete phylogeographic inference based on downsampled subsets of SARS-CoV-2 genomic sequences. In the lower-left corner, we report the distribution of the sizes of New York State clades. (**) For ease of visualization, the major state clade comprising 596 genomic sequences is not shown in the histogram.
