# Supplementary material for: Dispersal dynamics of SARS-CoV-2 lineages during the first epidemic wave in New York City
Source: PLoS Pathog. 2021 May 20;17(5):e1009571. doi: 10.1371/journal.ppat.1009571 (PMC8136714; doi:10.1371/journal.ppat.1009571)
Supplement: S2 Fig — On these maps schematizing the outcome of each replicated continuous phylogeographic analysis (Fig 2), we report the number of lineage dispersal events inferred among (arrows) and within (transparent grey circles) NYC boroughs, with both measures being averaged over 1,000 posterior trees sampled from each posterior distribution. Base layer for the maps has been obtained from https://www.census.gov. (PDF) [file ppat.1009571.s002.pdf]

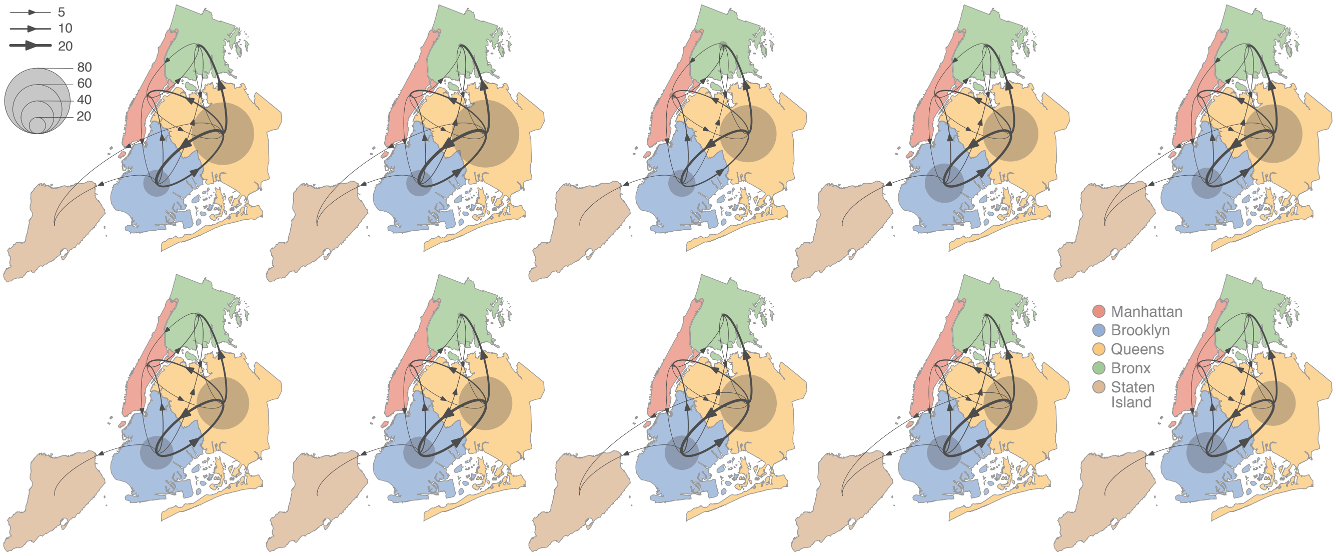

Figure S2. Schematic overview of the continuous phylogeographic analyses of the main SARS-CoV-2 clade circulating in New York City (NYC) during the first epidemic wave. On these maps schematizing the outcome of each replicated continuous phylogeographic analysis (Fig. 2), we report the number of lineage dispersal events inferred among (arrows) and within (transparent grey circles) NYC boroughs, with both measures being averaged over 1,000 posterior trees sampled from each posterior distribution. Base layer for the maps has been obtained from https://www.census.gov.
