# Supplementary material for: Dispersal dynamics of SARS-CoV-2 lineages during the first epidemic wave in New York City
Source: PLoS Pathog. 2021 May 20;17(5):e1009571. doi: 10.1371/journal.ppat.1009571 (PMC8136714; doi:10.1371/journal.ppat.1009571)
Supplement: S3 Fig — Contrary to the analyses reported in Fig 2 and obtained using the relaxed random walk (RRW) model, these alternative analyses were obtained using the relaxed directional random walk (RDRW) diffusion model, and led to phylogeographic reconstructions highly similar to the ones obtained with the RRW model (Fig 2). With this alternative RDRW diffusion model, maximum clade credibility (MCC) trees have, on average, fewer than 2% of their branches associated with significant latitudinal trends and fewer than 2% of their branches associated with significant longitudinal trends. Furthermore, RDRW analyses did not infer any global directional trend. As in Fig 2, each map corresponds to a distinct replicated phylogeographic inference based on a random subset of genomic sequences obtained after having subsampled NYC boroughs according to their cumulative number of new hospitalizations until the most recent sample (see the Methods section for details). For each replicated analysis, we mapped the maximum clade credibility (MCC) tree and overall 80% highest posterior density (HPD) regions reflecting the uncertainty related to the Bayesian phylogeographic inference. MCC trees and 80% HPD regions are based on 1,000 trees sampled from each post burn-in posterior distribution. Dispersal direction (anti-clockwise) of viral lineages is indicated by the edge curvature, MCC tree nodes are colored according to their time of occurrence, and 80% HPD regions were computed for successive time layers and then superimposed using the same color scale reflecting time. Base layer for the maps has been obtained from https://www.census.gov. (PDF) [file ppat.1009571.s003.pdf]

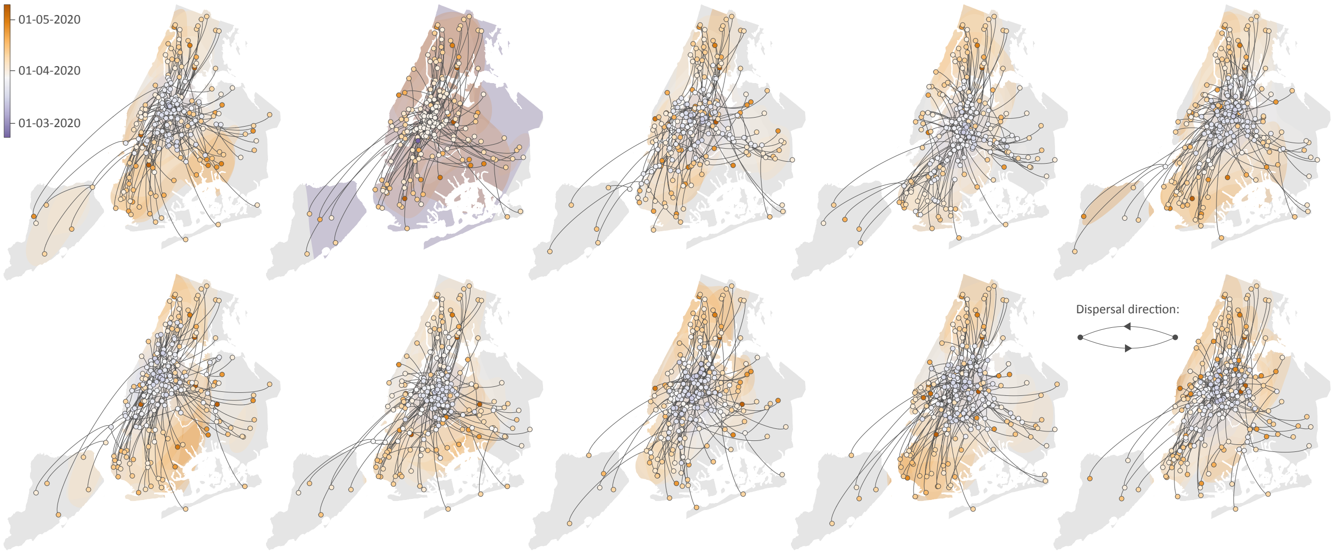

Figure S3. Alternative continuous phylogeographic analyses of the main SARS-CoV-2 clade circulating in New York City (NYC) during the first epidemic wave. Contrary to the analyses reported in Figure 2 and obtained using the relaxed random walk (RRW) model, these alternative analyses were obtained using the relaxed directional random walk (RDRW) diffusion model, and led to phylogeographic reconstructions highly similar to the ones obtained with the RRW model (Fig. 2). With this alternative RDRW diffusion model, maximum clade credibility (MCC) trees have, on average, fewer than 2% of their branches associated with significant latitudinal trends and fewer than 2% of their branches associated with significant longitudinal trends. Furthermore, RDRW analyses did not infer any global directional trend. As in Figure 2, each map corresponds to a distinct replicated phylogeographic inference based on a random subset of genomic sequences obtained after having subsampled NYC boroughs according to their cumulative number of new hospitalizations until the most recent sample (see the Methods section for details). For each replicated analysis, we mapped the maximum clade credibility (MCC) tree and overall 80% highest posterior density (HPD) regions reflecting the uncertainty related to the Bayesian phylogeographic inference. MCC trees and 80% HPD regions are based on 1,000 trees sampled from each post burn-in posterior distribution. Dispersal direction (anti-clockwise) of viral lineages is indicated by the edge curvature, MCC tree nodes are colored according to their time of occurrence, and 80% HPD regions were computed for successive time layers and then superimposed using the same color scale reflecting time. Base layer for the maps has been obtained from https://www.census.gov.
