# Supplementary material for: Dispersal dynamics of SARS-CoV-2 lineages during the first epidemic wave in New York City
Source: PLoS Pathog. 2021 May 20;17(5):e1009571. doi: 10.1371/journal.ppat.1009571 (PMC8136714; doi:10.1371/journal.ppat.1009571)
Supplement: S4 Fig — Each map corresponds to a distinct replicate discrete phylogeographic inference based on a random subset of genomic sequences obtained after having subsampled NYC boroughs according to their overall number of positive cases recorded until the most recent sample. As an alternative visualization to the average number of lineage transition events reported in Fig 3, we here report supported Markov jumps among NYC boroughs. Markov jumps are either supported by standard (A) or adjusted (B) Bayes factor values higher than 3, which correspond to positive support according to the scale of interpretation defined by Kass & Raftery (1995). Base layer for the maps has been obtained from https://www.census.gov. (PDF) [file ppat.1009571.s004.pdf]

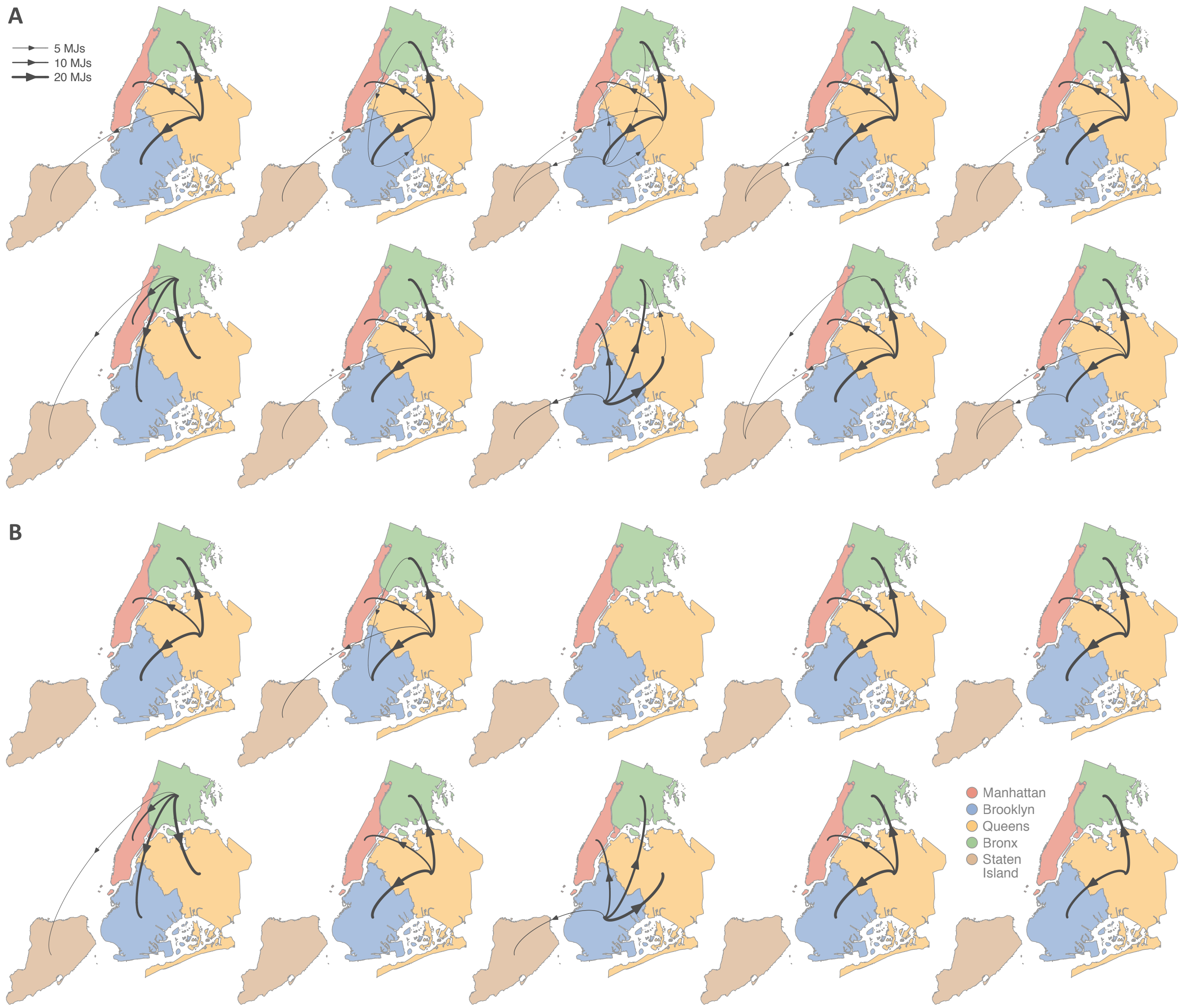

Figure S4. Well-supported Markov jumps inferred by discrete phylogeographic inferences dedicated to the main SARS-CoV-2 clade circulating in New York City (NYC) during the first epidemic wave. Each map corresponds to a distinct replicate discrete phylogeographic inference based on a random subset of genomic sequences obtained after having subsampled NYC boroughs according to their overall number of positive cases recorded until the most recent sample. As an alternative visualization to the average number of lineage transition events reported in Figure 3, we here report supported Markov jumps among NYC boroughs. Markov jumps are either supported by standard (A) or adjusted (B) Bayes factor values higher than 3, which correspond to positive support according to the scale of interpretation defined by Kass & Raftery (1995). Base layer for the maps has been obtained from https://www.census.gov.
