# Supplementary material for: Dispersal dynamics of SARS-CoV-2 lineages during the first epidemic wave in New York City
Source: PLoS Pathog. 2021 May 20;17(5):e1009571. doi: 10.1371/journal.ppat.1009571 (PMC8136714; doi:10.1371/journal.ppat.1009571)
Supplement: Attachment — Submitted filename: review1.pdf [file ppat.1009571.s005.pdf]

Review of MS #PPATHOGENS-D-21-00127
Dellicour et al.
“Dispersal dynamics of SARS-CoV-2 lineages during the first epidemic wave in New York City”

In this manuscript, the authors apply existing phylogeographic methods to SARS-CoV-2 genome sequences to obtain insight into patterns of virus transmission within the greater New York conurbation during the early phases of the COVID-19 pandemic. They first perform a single-tree analysis and then expand this to an analysis that incorporates phylogenetic uncertainty. In both of these, they employ both a continuous-space and then a discrete-space (metapopulation) model.

While the subject is interesting and important, it is impossible to adequately judge the significance of the conclusions because the methods are only perfunctorily characterized and described. In particular, the authors seek to justify their methods via a mere appeal to authority (saying, e.g., that the workflows are “standard” per other authors). There is nothing in phylogeographic analysis, however, that deserves to be considered standard. Indeed, even some common practices in this sort of analysis are questionable. Be this as it may, inasmuch as it is not even possible to *state* the conclusions of the paper without reference to the methods, it is essential that sufficent methodological detail be provided that the reader can understand the conclusions for what they are. Actually, more is required, since without an appreciation of the assumptions that have gone into the methods, one cannot judge which aspects of the conclusions are most strongly supported by the data, which are consequences of arbitrary but reasonable model assumptions, and which (if any) are consequences of invalid assumptions. Nor is it only the credibility of the conclusions that is influenced by the methods; methodological details shape one’s appreciation of the conclusions’ import as well. Finally, because the methodological details are so intimately connected with the data and the conclusions, it is essential that they be included in the paper in much more detail than they are currently given.

Here is an example. With respect to the continuous-space model, inasmuch as the model (including the prior) is isotropic, the effect will be to pull the physical locations attributed to phylogenetic nodes toward the center of the space. Accordingly, I suspect that the identification of the Greenpoint/Long Island City area as a locus of high phylogenetic centrality may be a more a consequence of its geographic centrality on the regional map than anything else. At any rate, this suspicion deserves exploration: how robust is that specific conclusion to varying priors?

I trust that, in responding to this and related criticism, the authors will feel no temptation to take refuge in nominally flat or so-called “uninformative” priors. As is well known, this is a canard: there is no such thing as an absolutely flat prior and even uniform priors contribute information to the posteriors. Nor should this be an instance where it will be sufficient to perform a bit of perfunctory fiddling with priors and other model assumptions, followed by verification that the conclusions remain superficially unchanged. Rather, the authors should ask themselves the question: Within the bounds of what would be considered permissible in a Bayesian analysis, how much prejudice against each of our conclusions must we build into the model to get a meaningfully different result? And also: As we build into the model increasing degrees of prejudice against our conclusions, in what ways do the latter change, either quantitatively and qualitatively? If the information in the data turns out to be so overwhelming that only extreme prejudice can suppress it, it is important to know this. On the other hand, if (as I suspect) it turns out that some aspects of the conclusions are highly sensitive to model assumptions, it is equally important to know it.

To get at these issues, a variety of incisive experiments with the model can be readily imagined. For example, does including Nassau or Westchester Counties, or northern New Jersey, with their large populations and strong economic ties to the city, affect the location of regions of high phylogenetic centrality? What happens if one drops Staten Island?

From this point of view, the discrete-space model is less obviously, but potentially equally, problematic. It is just as important to understand its assumptions in detail. For example, is it a perfectly generic, flexible migration model (with $n\,(n-1)$ parameters), or do covariates (perhaps population sizes, commuting data, etc.) constrain the model? The remarks above concerning the importance of priors and other model assumptions apply with equal force here. Indeed, such considerations are in a sense even more critical here than they were for the continuous-space model, since it may be more difficult to identify the consequences of arbitrary model assumptions in this more complex model.

Certain others of the model assumptions appear quite arbitrary. For example, how does one justify allowing localities within New York State to play a distinguished role not shared by regions of New Jersey and Connecticut that are potentially much more strongly connected to the city in all the ways that matter for transmission? Similarly, what is the magnitude of population movements into the city from more far-flung regions via the city's airports and intercity trains during this period, relative to those from the suburbs? I do not mean to suggest that it is strictly necessary that the authors expand the geographic scope of their study. Rather, my point is that one needs to investigate the extent to which geographical distinctions made in the analysis—distinctions which are respected neither by the host nor the virus—may be responsible for certain of the quantitative or qualitative conclusions.

Finally, I anticipate that, in the course of improving their analysis, the authors may develop hypotheses as to the causal mechanisms underlying certain of the patterns they observe. The paper would be much strengthened were they to follow up on these, using additional data as appropriate. After all, by their very nature, the highly flexible, generic models of the sort employed in this paper, can say little, if anything, about the true drivers of the patterns observed. They are best viewed, therefore, as sophisticated tools for exploratory data analysis and hypothesis generation.

Specific remarks

1. ll 80ff: The authors recognize that inadequate sampling (or perhaps overly narrow data-inclusion criteria) limit their ability to resolve clusters with substantial extent beyond the boundaries of the city proper. In view of this lack of resolution, it would be more straightforward to state it as I just have rather than as a positive result ("Continuous phylogeographic reconstructions highlight that most peripheral samples are directly connected to the NYC area rather than clustered together (Fig. S2). In other words, samples collected outside NYC likely correspond to distinct dispersal events originating from the city area.") qualified by a caveat ("However, we acknowledge that areas surrounding NYC were relatively less densely sampled than NYC boroughs, which might lead to an underestimation of the circulation of viral lineages in the surrounding areas."). It seems that one could, with equal evidence and at least as great plausibility, put it the other way around.

2. l 97: "…according to their relative importance during the epidemiological phase under investigation". The authors assume that relative importance is adequately measured by proportion of hospitalizations. Are there alternative metrics that might be equally pertinent? What impacts would bias in hospitalization rates among boroughs—which have markedly different income and employment profiles, and therefore presumably different rates of insurance coverage—have on the analysis?

3. l 128: In what sense is the discrete model not spatially explicit? I interpreted this model as being what an ecologist would call a spatially-explicit metapopulation model. If I did so incorrectly, this is more testimony to the need for better description of the models.

4. Why was downsampling necessary?

5. The authors highlight the importance of heterogeneities in sampling as potentially consequential to their conclusions. They ignore, however, the many other model assumptions (most of them left unmentioned

for the reader to guess at according to the degree of their experience and insight) about the magnitudes and directions of host movement fluxes, transmission rates, recovery rates, etc.

6. ll 203ff: The description of the MCMC protocols begs the question as to the extent these were checked for adequacy. It is generically the case that the algorithmic parameters used by previous studies will be inadequate, or at least sub-optimal, for a given, new MCMC analysis, even when the former appear similar in some ways to the latter. How did the authors determine that their algorithmic choices (number of MCMC iterations, number of chains, length of burn-in period, etc.) achieved convergence to the posterior and allowed adequate sampling therefrom? How did they verify convergence? How did they establish mixing? What specific MCMC variant was employed?

7. l 248: The authors assumed a strict molecular clock. To what extent did this strong prior affect their conclusions?
