# Supplementary material for: Dispersal dynamics of SARS-CoV-2 lineages during the first epidemic wave in New York City
Source: PLoS Pathog. 2021 May 20;17(5):e1009571. doi: 10.1371/journal.ppat.1009571 (PMC8136714; doi:10.1371/journal.ppat.1009571)
Supplement: Attachment — Submitted filename: Response_to_Reviewers.pdf [file ppat.1009571.s006.pdf]

Dr. Simon Dellicour

Spatial Epidemiology Lab
Université Libre de Bruxelles
50 avenue F.D. Roosevelt
1050 Bruxelles, Belgium

Rega Institute
KU Leuven – University of Leuven
Herestraat 49
3000 Leuven, Belgium

Brussels (B), 27.03.2021

Manuscript re-submission (PPATHOGENS-D-21-00127): "Dispersal dynamics of SARS-CoV-2 lineages during the first epidemic wave in New York City" by Simon Dellicour, Samuel L. Hong, Bram Vrancken, Antoine Chaillon, Mandev Gill, Matthew Maurano, Paul Zappile, Christian Marier, Gordon Harkins, Guy Baele, Ralf Duerr, and Adriana Heguy.

Dear Editors,

On behalf of all authors, I am pleased to re-submit to *PLoS Pathogens* a revised version of our manuscript entitled "Dispersal dynamics of SARS-CoV-2 lineages during the first epidemic wave in New York City" (PPATHOGENS-D-21-00127).

We would like to express our gratitude to the editorial team and the referees for the constructive and relevant comments on the previous version of our manuscript. We have now revised the manuscript in response to the reviewers' comments (see below for a point-by-point response to all comments) and hope it can now be found suitable for publication in *PLoS Pathogens*.

Yours sincerely,

Simon Dellicour

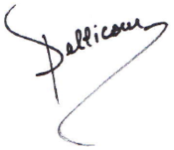

Part I – Summary

Please use this section to discuss strengths/weaknesses of study, novelty/significance, general execution and scholarship.

Reviewer #1: This is a nice paper, about using phylogeographic methods to uncover the transmission pattern of SARS-CoV-2 in New York. The authors use a set of around 800 sequences and subsample multiple times to try and mitigate spatial sampling bias. The methods used - the discrete and continuous phylogeographic methods are well chosen and executed for the data and the limitations of the methods are clearly discussed.

However, this is paper focuses on spread of one main introduction to NY, and according to the data shown this is the main clade in wave 1. Presumably the other introductions didn't seed (sub)lineages long lived enough to sample and analyse, and it would be useful to include some more on these so that the difference between the successful and unsuccessful (sub)lineages can be more easily shown.

Answer: Thank you very much for your positive feedback on our work. We agree with the Reviewer that, in the previous version of the manuscript, our focus was solely on the main introduction event as this represented the largest outbreak occurring at the time and we neglected to present the information for the remaining introduction events that led to smaller outbreaks and clades that we detected circulating in the study area. To address this issue, we have moved the previous Figure S2 to the main text, and also added a histogram to Figure S1 showing the distribution of clade sizes in New York State resulting from distinct introduction events (Fig. R1).

As illustrated in this histogram, most introduction events into New York State resulted in notably small clades of SARS-CoV-2 genomic sequences sampled across the study area, and the majority (69%) were only identified in unique samples (singletons). As the Reviewer correctly presumes, the majority of the remaining introductions likely did not seed lineages long live enough to sample and analyse.

Moving the previous Figure S2 (now Figure 1) to the main text now allows us to put more emphasis on the overall phylogeographic pattern that arises from all introduction events considered together. In addition, this figure also includes a visualization of the temporal distribution of the estimated time to the most recent common ancestor (TMRCA) of each circulating clade resulting from a distinct introduction event into New York State.

Reviewer #2: [Please see the attached PDF, which may have some typographical elements the following plain-text version misses.] In this manuscript, the authors apply existing phylogeographic methods to SARS-CoV-2 genome sequences to obtain insight into patterns of virus transmission within the greater New York conurbation during the early phases of the COVID-19 pandemic. They first perform a single-tree analysis and then expand this to an analysis that incorporates phylogenetic uncertainty. In both of these, they employ both a continuous-space and then a discrete-space (metapopulation) model.

While the subject is interesting and important, it is impossible to adequately judge the significance of the conclusions because the methods are only perfunctorily characterized and described. In particular, the authors seek to justify their methods via a mere appeal to authority (saying, e.g., that the workflows are "standard" per other authors). There is nothing in phylogeographic analysis, however, that deserves to be considered standard. Indeed, even some common practices in this sort of analysis are questionable. Be this as it may, inasmuch as it is not even possible to *state* the conclusions of the paper without reference to the methods, it is essential that sufficient methodological detail be provided that the reader can understand the conclusions for what they are. Actually, more is required, since without an appreciation of the assumptions that have gone into the methods, one cannot judge which aspects of the conclusions are most strongly supported by the data, which are consequences of arbitrary but reasonable model assumptions, and which (if any) are consequences of invalid assumptions. Nor is it only the credibility of the conclusions that is influenced by the methods; methodological details shape one's appreciation of the conclusions' import as

well. Finally, because the methodological details are so intimately connected with the data and the conclusions, it is essential that they be included in the paper in much more detail than they are currently given.

Answer: Thank you for your helpful comment pointing out that insufficient information was provided on the methodological details and the implicit assumptions of the various analytical methods we employed so that the reader may better understand the conclusions for what they are. Developed more than ten years ago, these two methods (discrete and continuous phylogeographic inferences) have been extensively employed in the field of molecular epidemiology and beyond. We therefore incorrectly considered it to be beyond the scope of our study to describe such well-established methodologies in too much detail in this manuscript. We acknowledge that an adequate description of these two methods was lacking, and in response, we have now revised the text accordingly to address this concern.

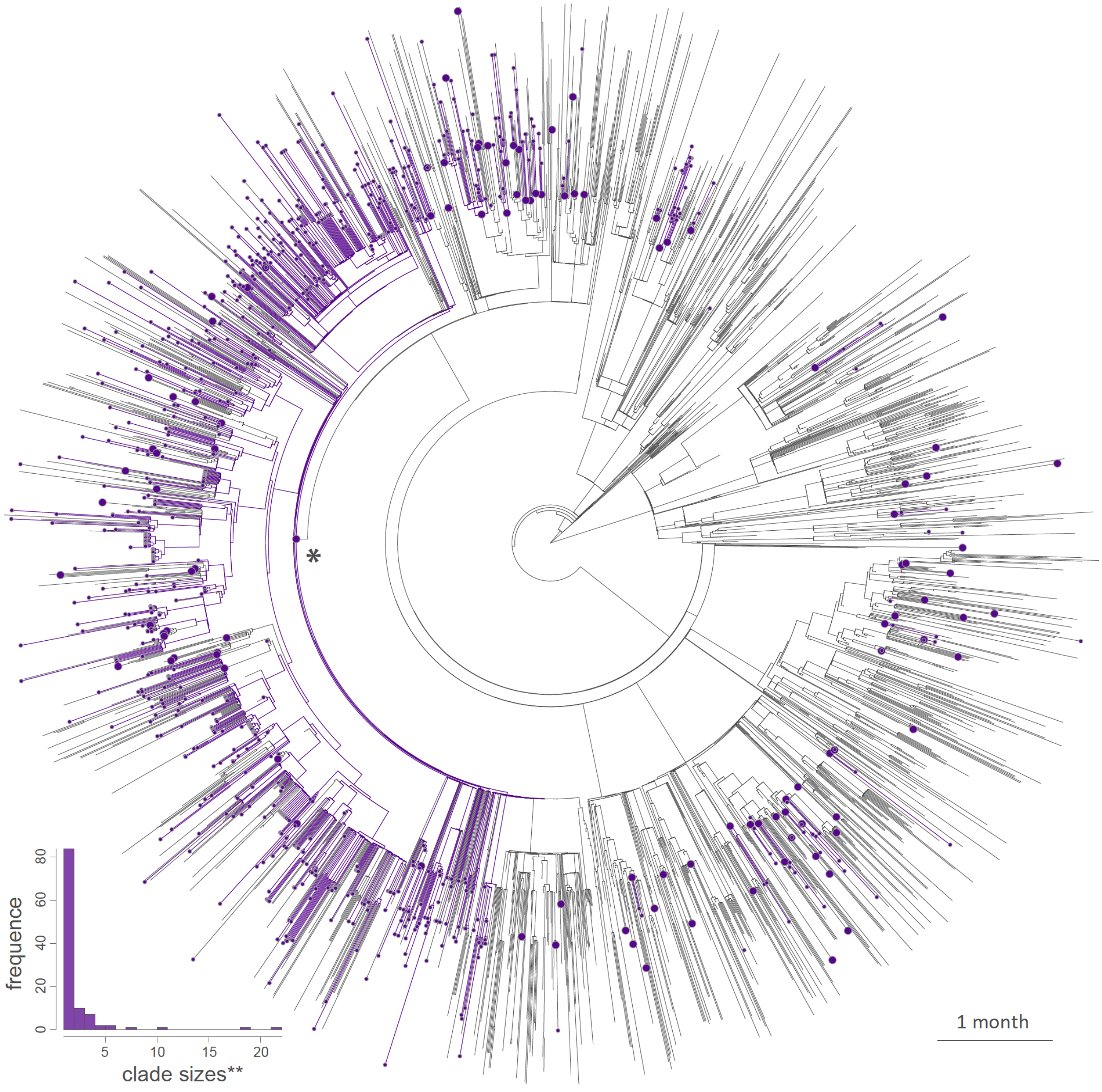

Figure R1 (Figure S1 in our study). Time-scaled phylogenetic tree in which we identified SARS-CoV-2 phylogenetic clades introduced in New York State during the first epidemic wave. […]. In the lower-left corner, we report the distribution of the sizes of New York State clades. (**) For ease of visualization, the major state clade comprising 596 genomic sequences is not shown in the histogram.

Here is an example. With respect to the continuous-space model, in as much as the model (including the prior) is isotropic, the effect will be to pull the physical locations attributed to phylogenetic nodes toward the center of the space. Accordingly, I suspect that the identification of the Greenpoint/Long Island City area as a locus of high phylogenetic centrality may be more a consequence of its geographic centrality on the regional map than anything else. At any rate, this suspicion deserves exploration: how robust is that specific conclusion to varying priors?

I trust that, in responding to this and related criticism, the authors will feel no temptation to take refuge in nominally flat or so-called "uninformative" priors. As is well known, this is a canard: there is no such thing as an absolutely flat prior and even uniform priors contribute information to the posteriors. Nor should this be an instance where it will be sufficient to perform a bit of perfunctory fiddling with priors and other model assumptions, followed by verification that the conclusions remain superficially unchanged. Rather, the authors should ask themselves the question: Within the bounds of what would be considered permissible in a Bayesian analysis, how much prejudice against each of our conclusions must we build into the model to get a meaningfully different result? And also: As we build into the model increasing degrees of prejudice against our conclusions, in what ways do the latter change, either quantitatively and qualitatively? If the information in the data turns out to be so overwhelming that only extreme prejudice can suppress it, it is important to know this. On the other hand, if (as I suspect) it turns out that some aspects of the conclusions are highly sensitive to model assumptions, it is equally important to know it.

Answer: This is an important point. The relaxed random walk (RRW) diffusion model used for the continuous phylogeographic reconstructions is an anisotropic random walk model (Pybus *et al*. 2012, PMID: 22927414), which has demonstrated the ability to infer non-central locations of internal nodes. However, in the RRW model, longitude and latitude are treated as continuous traits modelled according to a time-scaled mixture of Brownian diffusion processes with a zero-mean displacement along each phylogenetic branch, and it has been recognized that in some situations it can inadequately model a diffusion process characterized by directional trends (Gill *et al*. 2017, PMID: 27798403). To overcome these limitations in the RRW model, Gill and colleagues have recently developed a relaxed directional random walk (RDRW) that relaxes the assumption of a zero-mean displacement along phylogenetic branches (Gill *et al*. 2017). To address the legitimate concerns of the Reviewer and assess the sensitivity of the results to our modelling assumptions, we re-ran the ten replicated continuous phylogeographic analyses using the RDRW instead of the RRW model, and found that the RDRW model yielded highly similar phylogeographic reconstructions (Fig. R2). With this alternative diffusion model that allows for branch-specific directional trends, we found that the maximum clade credibility (MCC) trees have, on average, fewer than 2% of their branches associated with significant latitudinal trends and fewer than 2% of their branches associated with significant longitudinal trends. Furthermore, RDRW analyses did not reveal any global directional trends. We now integrate these aspects within the text and present the RDRW reconstructions in Supplementary Information.

To get at these issues, a variety of incisive experiments with the model can be readily imagined. For example, does including Nassau or Westchester Counties, or northern New Jersey, with their large populations and strong economic ties to the city, affect the location of regions of high phylogenetic centrality? What happens if one drops Staten Island?

Answer: We do not have sufficient numbers of samples to perform all of these alternative tests. However, along with the additional RDRW analyses described above, we have now re-performed the ten replicated continuous phylogeographic analyses after having discarded sequences sampled in Staten Island. We followed this suggestion made by the Reviewer to illustrate that our continuous phylogeographic reconstructions are not impacted when discarding those genomic sequences sampled from a relatively more

remote area. As illustrated in Figure R3, the general pattern of inferred lineage dispersal history is not altered by this alternative sub-selection of sampled genomes.

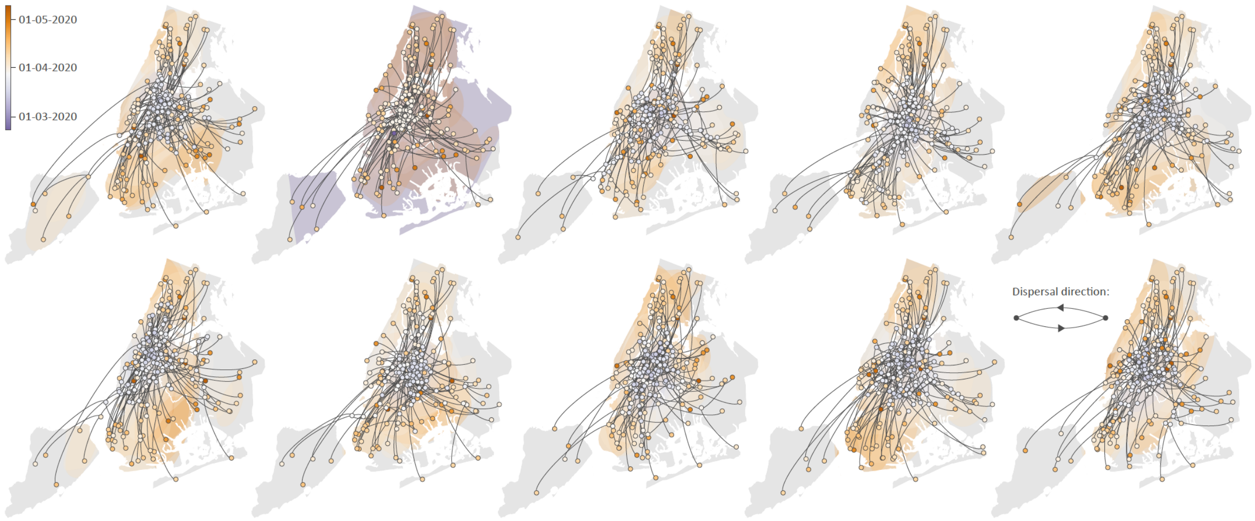

Figure R2 (Figure S3 in our study). Alternative spatially-explicit phylogeographic analyses of the main SARS-CoV-2 clade circulating in New York City (NYC) during the first epidemic wave. Contrary to the analyses reported in Figure 2 and obtained using the relaxed random walk (RRW) model, these alternative analyses were obtained using the relaxed directional random walk (RDRW) diffusion model. As in Figure 2, each map corresponds to a distinct replicated phylogeographic inference based on a random subset of genomic sequences obtained after having subsampled NYC boroughs according to their cumulated number of new hospitalizations until the most recent sample (see the Methods section for details). For each replicated analysis, we mapped the maximum clade credibility (MCC) tree and overall 80% highest posterior density (HPD) regions reflecting the uncertainty related to the Bayesian phylogeographic inference. MCC trees and 80% HPD regions are based on 1,000 trees sampled from each post burn-in posterior distribution. Dispersal direction (anti-clockwise) of viral lineages are indicated by the edge curvature, MCC tree nodes are colored according to their time of occurrence, and 80% HPD regions were computed for successive time layers and then superimposed using the same color scale reflecting time.

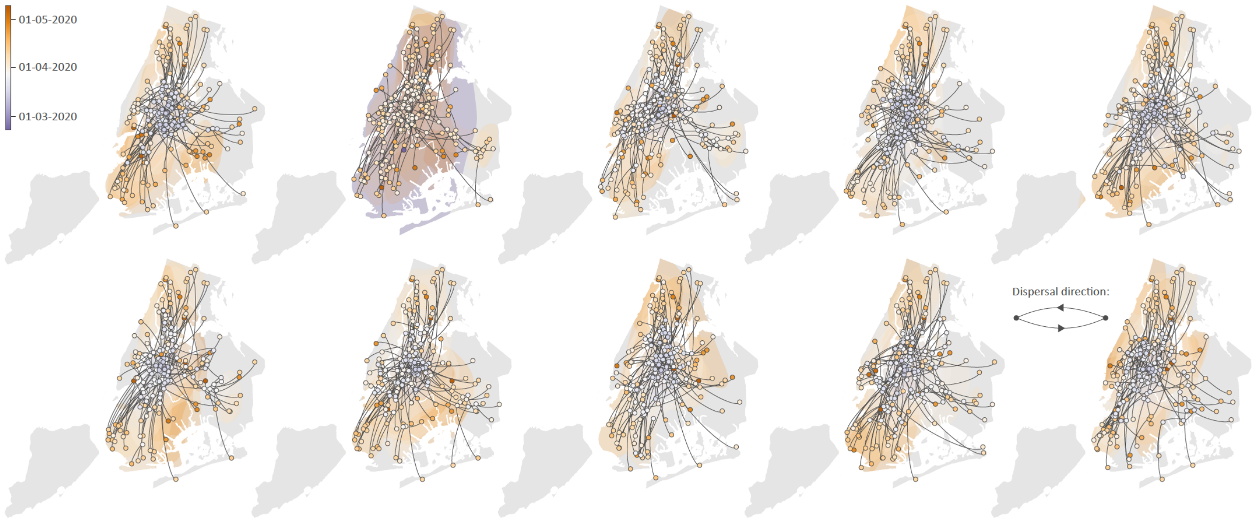

Figure R3. Spatially-explicit phylogeographic reconstructions obtained when excluding SARS-CoV-2 genomic sequences sampled in Staten Island. Each map corresponds to a distinct replicated phylogeographic inference based on a random subset of genomic sequences obtained after having subsampled NYC boroughs according to their cumulated number of new hospitalizations until the most recent sample (see the Methods section for details). For each replicated analysis, we mapped the maximum clade credibility (MCC) tree and overall 80% highest posterior density (HPD) regions reflecting the uncertainty related to the Bayesian phylogeographic inference. MCC trees and 80% HPD regions are based on 1,000 trees sampled from each post burn-in posterior distribution. Dispersal direction (anti-clockwise) of viral lineages are indicated by the edge curvature, MCC tree

nodes are colored according to their time of occurrence, and 80% HPD regions were computed for successive time layers and then superimposed using the same color scale reflecting time.

From this point of view, the discrete-space model is less obviously, but potentially equally, problematic. It is just as important to understand its assumptions in detail. For example, is it a perfectly generic, flexible migration model (with $n\,(n-1)$ parameters), or do covariates (perhaps population sizes, commuting data, etc.) constrain the model? The remarks above concerning the importance of priors and other model assumptions apply with equal force here. Indeed, such considerations are in a sense even more critical here than they were for the continuous-space model, since it may be more difficult to identify the consequences of arbitrary model assumptions in this more complex model.

Answer: As now explicitly stated in the text, the discrete phylogeographic approach is a discrete character mapping analysis based on a discrete diffusion model that is not constrained by external covariates, and not a type of metapopulation model (see also our answer to the related comment of the Reviewer below). The discrete phylogeographic analysis is based on a stochastic diffusion process modeled according to a continuous-time Markov chain here characterized by a matrix of asymmetrical transition rates among sampling locations (Lemey *et al*. 2009, PMID: 19779555; Faria *et al*. 2011, PMID: 22440846).

Certain others of the model assumptions appear quite arbitrary. For example, how does one justify allowing localities within New York State to play a distinguished role not shared by regions of New Jersey and Connecticut that are potentially much more strongly connected to the city in all the ways that matter for transmission? Similarly, what is the magnitude of population movements into the city from more far-flung regions via the city's airports and intercity trains during this period, relative to those from the suburbs? I do not mean to suggest that it is strictly necessary that the authors expand the geographic scope of their study. Rather, my point is that one needs to investigate the extent to which geographical distinctions made in the analysis - distinctions which are respected neither by the host nor the virus - may be responsible for certain of the quantitative or qualitative conclusions.

Answer: We agree with the Reviewer that the discretization of the sampling area will always remain arbitrary, and will only allow a reconstruction of the dispersal history of lineages among sampled locations, which is now explicitly stated in the text when introducing this model. In the present study, we specifically used a discrete phylogeographic approach to investigate the dispersal dynamic of SARS-CoV-2 lineages among NYC boroughs, which corresponds to an arbitrary yet geographically and administratively recognized subdivision of the city. Such a discrete phylogeographic approach is precisely useful when the aim is to analyze exchange of viral lineages among well-defined geographic entities. On the other hand, the use of a continuous phylogeographic approach does not require a preliminary subdivision of the study area into discrete entities, and thus corresponds to a different but complementary approach to the discrete one. In the present study, we used both approaches to get a more complete and fine-scale picture of the SARS-CoV-2 circulation within NYC. Of note, we decided to move previous Figure 3 to Supplementary Information (now Figure S2) in order to avoid some confusion with the discrete phylogeographic reconstructions, which correspond, by definition, to the most appropriate approach to investigate dispersal transition of viral lineages among discrete locations.

Finally, I anticipate that, in the course of improving their analysis, the authors may develop hypotheses as to the causal mechanisms underlying certain of the patterns they observe. The paper would be much strengthened were they to follow up on these, using additional data as appropriate. After all, by their very nature, the highly flexible, generic models of the sort employed in this paper, can say little, if anything, about the true drivers of the patterns observed. They are best viewed, therefore, as sophisticated tools for exploratory data analysis and hypothesis generation.

Answer: Our study provides analyses of the SARS-CoV-2 dispersal dynamics across the study area in the first phase of the COVID-19 epidemic when New York State was the epicentre of the global pandemic. Our aim was to determine whether using the available spatial and genomic data, we could reliably infer - using well established Bayesian probabilistic modeling methods - the historical dispersal and transmission dynamics in NY. In our analyses, we have gone to great lengths to account for any sampling or methodological biases that we identified. Hopefully now that we have, as requested by the Reviewer, provided much more information about the methods used and their underlying assumptions the Reviewer can more readily assess the statistical reliability of the conclusions we draw from the reconstructed dispersal patterns we report. Our findings suggest several hypotheses that could be tested with the appropriate data. However, identifying these drivers was beyond the scope of this investigation given the limited data available to us at the time (a situation that remains largely unchanged). We have now extended the related part of the Discussion section on the reliability of our inferences and the potential external factors that might have shaped the patterns we recovered.

Part II – Major Issues: Key Experiments Required for Acceptance

Please use this section to detail the key new experiments or modifications of existing experiments that should be absolutely required to validate study conclusions.

Generally, there should be no more than 3 such required experiments or major modifications for a "Major Revision" recommendation. If more than 3 experiments are necessary to validate the study conclusions, then you are encouraged to recommend "Reject".

Reviewer #1: (No Response)

Reviewer #2: See Summary above. Also:

1. ll 80ff: The authors recognize that inadequate sampling (or perhaps overly narrow data-inclusion criteria) limit their ability to resolve clusters with substantial extent beyond the boundaries of the city proper. In view of this lack of resolution, it would be more straightforward to state it as I just have rather than as a positive result ("Continuous phylogeographic reconstructions highlight that most peripheral samples are directly connected to the NYC area rather than clustered together (Fig. S2). In other words, samples collected outside NYC likely correspond to distinct dispersal events originating from the city area.") qualified by a caveat ("However, we acknowledge that areas surrounding NYC were relatively less densely sampled than NYC boroughs, which might lead to an underestimation of the circulation of viral lineages in the surrounding areas."). It seems that one could, with equal evidence and at least as great plausibility, put it the other way around.

Answer: We fully agree with the remark of the Reviewer and have edited these sentences accordingly.

2. l 97: "...according to their relative importance during the epidemiological phase under investigation". The authors assume that relative importance is adequately measured by proportion of hospitalizations. Are there alternative metrics that might be equally pertinent? What impacts would bias in hospitalization rates among boroughs - which have markedly different income and employment profiles, and therefore presumably different rates of insurance coverage - have on the analysis?

Answer: We agree with the Reviewer that the number of new hospitalizations could indeed be to some extent biased, which is now explicitly recognized in the text. However, we argue that, in order to reflect the general evolution of the epidemic or local incidence, it should in theory constitute a better proxy than an

indicator based on the number of positive cases, because the latter is directly impacted by the testing effort and strategy that are both evolving over time.

3. l 128: In what sense is the discrete model not spatially explicit? I interpreted this model as being what an ecologist would call a spatially-explicit metapopulation model. If I did so incorrectly, this is more testimony to the need for better description of the models.

Answer: The discrete phylogeographic method used in our study is not based on a metapopulation model but on a discrete character mapping approach. This method is classified as "discrete in space" because it does not treat the study area as a continuous space. As now also explicitly stated in the text: "*While the discrete phylogeographic method requires the preliminary delimitation of discrete locations exchanging viral lineages (and hence cannot estimate ancestral locations that are not part of this predefined set), the continuous method is based on a spatially-explicit in which internal nodes can occur in unsampled locations. This continuous method employs an anisotropic relaxed random walk (RRW) [Pybus et al. 2012, PMID: 22927414] and can be considered as a continuous character mapping approach treating longitude and latitude as two separate continuous traits.*"

4. Why was downsampling necessary?

Answer: The downsampling was necessary to avoid performing our continuous and discrete phylogeographic reconstructions on a data set that resulted from a heterogeneity in the sampling effort that does not adequately reflect differences in local COVID-19 incidence (see also our above answer to the comment on the metric used as relatively reliable proxy of the incidence). Failing to correct for such sampling bias can potentially lead to artifactual outcomes in the phylogeographic reconstructions (De Maio *et al*. 2015, PMID: 26267488; Kalkauskas *et al*. 2021, PMID: 33406072). This element has now been made more explicit in the text.

5. The authors highlight the importance of heterogeneities in sampling as potentially consequential to their conclusions. They ignore, however, the many other model assumptions (most of them left unmentioned for the reader to guess at according to the degree of their experience and insight) about the magnitudes and directions of host movement fluxes, transmission rates, recovery rates, etc.

Answer: If not treated, heterogeneity in sampling effort could indeed lead to some artifactual results. As stated above, this aspect precisely motivated us to adopt a procedure based on a repeated downsampling approach. The diffusion model behind the discrete character mapping approach used in our study is based on the assumption of a transition rate matrix jointly inferred along the phylogenetic tree connecting sampled genomic sequences. In the end, the discrete phylogeographic reconstructions are thus only based on the genetic information contained in genomic sequences.

6. ll 203ff: The description of the MCMC protocols begs the question as to the extent these were checked for adequacy. It is generically the case that the algorithmic parameters used by previous studies will be inadequate, or at least sub-optimal, for a given, new MCMC analysis, even when the former appear similar in some ways to the latter. How did the authors determine that their algorithmic choices (number of MCMC iterations, number of chains, length of burn-in period, etc.) achieved convergence to the posterior and allowed adequate sampling therefrom? How did they verify convergence? How did they establish mixing? What specific MCMC variant was employed?

Answer: The convergence and proper mixing of MCMC algorithms were checked using the program Tracer (Rambaut *et al*. 2017, PMID: 29718447; Fig. R4), which also allows for the computation of effective sample size (ESS) values to assess if a sufficient number of independent samples have been collected from

the posterior distribution, for all continuous parameters. As now also explicitly stated in the text, we used a Metropolis-Hastings algorithm.

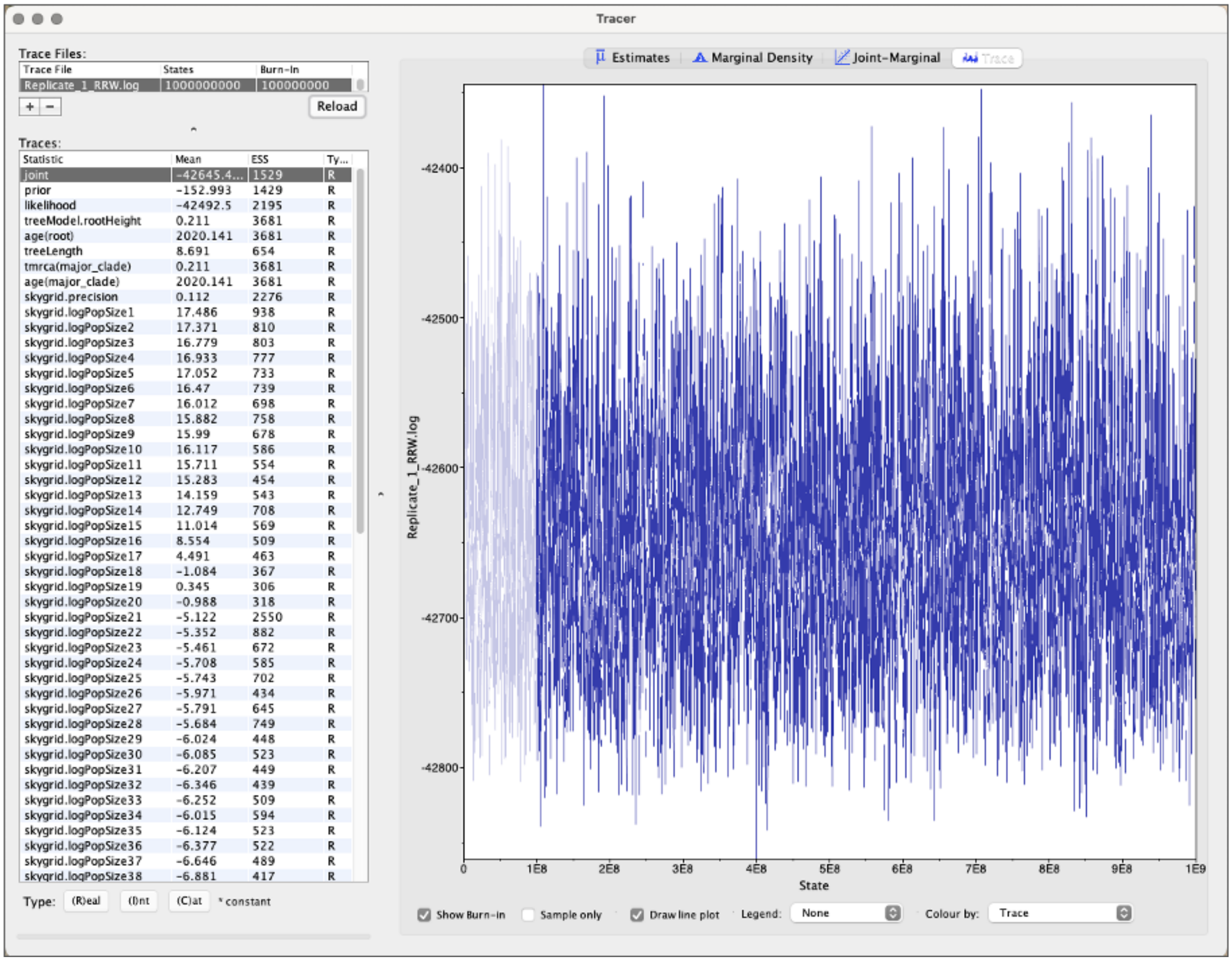

Figure R4. Use of the program Tracer 1.7 to assess the convergence and proper mixing of MCMC algorithms (here for the first replicate continuous phylogeographic analysis). In addition to visualizing the trace and posterior distribution associated with inferred parameters, the program computed the estimated sample size (ESS) value associated with each of these parameters in order to establish if the MCMC has sampled enough uncorrelated posterior trees.

7. l 248: The authors assumed a strict molecular clock. To what extent did this strong prior affect their conclusions?

Answer: The choice of a strict molecular clock model arises from the specification of an evolutionary rate fixed to an independent estimate to accommodate the lack of temporal signal associated with the subsets of genomic sequences analyzed in our study (confirmed using the root-to-tips regression approach implemented in TempEst; Rambaut *et al.* 2016, PMID: 27774300). Since the considered set of genomic sequences did not allow to calibrate a molecular clock model due to the lack of temporal signal, there was no point in specifying a more parametric model such as a relaxed clock model. Since our conclusions are mainly based on the inference of lineage dispersal events in themselves (and not on their specific timing), we can reasonably assume that the constrained choice of a strict molecular model does not have any notable impact on the dispersal patterns discussed in our study.

Part III – Minor Issues: Editorial and Data Presentation Modifications

Please use this section for editorial suggestions as well as relatively minor modifications of existing data that would enhance clarity.

Reviewer #1: There are a few minor issues that I noticed:

Data set - GISAID (NY NYUMC2-929)
Is this now public accessible data and would it make sense to list the EPI_ISL in supplementary (I notice that you have the sequences info in GitHub though).

Answer: We confirm that all the sequences have been uploaded on GISAID and that their associated IDs are available on the GitHub repo of this study.

Also, although you mention this in the methods text, I wonder if it would be useful to show something like a bar graph / table with the numbers of sequences from each discrete place per epi-week?

Answer: In part to address this comment, we decided to move the previous Figure S2 to the main text (now labelled Figure 1). This figure includes a graphic displaying the temporal density of sampled sequences over time.

Line 84 - I wonder if you are mostly looking at the one big clade; would you miss lineages spreading differently in the areas surrounding NYC especially if they are seeded from outside NY area (or outside US)?

Answer: We only focused on the big clade for performing joint continuous and discrete phylogeographic analyses of the dispersal history and dynamic of SARS-CoV-2 lineages within the NYC area. In our preliminary analysis aiming at investigating the overall pattern of phylogeographic connections among NYC and surrounding areas, we based our analysis on all available sequences for the time period under investigation. However, we admit that there was not enough emphasis on this analysis previously presented in Supplementary Information. Following the Reviewer's comment, we decided to move the outcome of this preliminary more comprehensive phylogeographic analysis to the main text (new Figure 1). To answer to the specific concern raised by the Reviewer, we have now completed the text as follows: "*While this phylogeographic pattern would indicate that samples collected outside NYC likely correspond to distinct dispersal events originating from the city area, we however acknowledge that areas surrounding NYC were relatively less densely sampled than NYC boroughs, which might lead to an underestimation of the circulation of viral lineages in the surrounding areas. A denser sampling in areas surrounding NYC could help clarifying the actual extent of local dispersal of viral lineages in these areas.*"

Line 96-101 approximate - Can you indicate the range of numbers of sequences in the subsets here.

Answer: The number of sequences subsampled by borough is detailed in the Methods section: "*The downsampled data sets comprised the following number of sequences: n = 17 (Manhattan), 31 (Brooklyn), 36 (Queens), 26 (The Bronx), and 5 (Staten Island)*". A priori, we would prefer to leave this sentence in the Methods section because it follows a detailed description of the rationale behind the selection of the number of genomic sequences to subsample from each area.

Figures 1-3: Doing the 10 replicates is a good idea, but is there an easier way to summarise all the 10 networks on maps? For example for Figures 2 & 3 - put all 10 on the same map with line thickness and transparency being the sum of the individual replicate lines? And then put all 10 replicates individually in the supplementary? and then you could put the summary maps side by side in the main text. Also for figure 1 - you could just add all the networks to one map and add transparency to the lines (so that multiple links in almost the same places will re-inforce)?

Answer: We believe that presenting a workflow with replicated analyses based on randomly subsampled data sets is a methodological interest of our study. In that context, highlighting the consistency of our results as well as the few differences among those replicates is illustrative of the importance to consider such an approach to deal with and assess the potential impact of heterogeneity in sampling effort. For this conceptual reason, as well as to avoid potential confusion for the readership, we would prefer to keep distinct visualizations for our replicated continuous/discrete phylogeographic analyses.
