# Supplementary material for: Dispersal dynamics of SARS-CoV-2 lineages during the first epidemic wave in New York City
Source: PLoS Pathog. 2021 May 20;17(5):e1009571. doi: 10.1371/journal.ppat.1009571 (PMC8136714; doi:10.1371/journal.ppat.1009571)
Supplement: Attachment — Submitted filename: Response_to_Reviewers.pdf [file ppat.1009571.s007.pdf]

Dr. Simon Dellicour

Spatial Epidemiology Lab
Université Libre de Bruxelles
50 avenue F.D. Roosevelt
1050 Bruxelles, Belgium

Rega Institute
KU Leuven – University of Leuven
Herestraat 49
3000 Leuven, Belgium

Brussels (B), 16.04.2021

Manuscript re-submission (PPATHOGENS-D-21-00127): “Dispersal dynamics of SARS-CoV-2 lineages during the first epidemic wave in New York City” by Simon Dellicour, Samuel L. Hong, Bram Vrancken, Antoine Chaillon, Mandev Gill, Matthew Maurano, Paul Zappile, Christian Marier, Gordon Harkins, Guy Baele, Ralf Duerr, and Adriana Heguy.

Dear Editors,

On behalf of all authors, I am pleased to re-submit to *PLoS Pathogens* a second revised version of our manuscript entitled “Dispersal dynamics of SARS-CoV-2 lineages during the first epidemic wave in New York City” (PPATHOGENS-D-21-00127). We have now revised the manuscript in response to the remaining editor’s comments (see below for a point-by-point response to all comments) and hope it can now be found suitable for publication in *PLoS Pathogens*.

Yours sincerely,

Simon Dellicour

Thank you for your revision. In the interest of expediting a decision, this was handled at the editorial level. Your efforts to address these comments are much appreciated. There is one more issue that should be addressed. Reviewer 2 asked for descriptions of the assumptions, priors, and MCMC properties (e.g. length, sampling, burn in) for the analyses. It appears that they were added for the fixed tree analysis, but not for the secondary analyses. Unless I have missed it in the revised manuscript, please add this information in a second revision. Once that is complete, I should be able to render a formal editorial acceptance.

Answer: MCMC properties were indeed missing for the replicated phylogeographic analyses and the related text has now been updated as follows: "*For each downsampled data set, we performed both a continuous and a discrete phylogeographic analysis using the respective diffusion models implemented in BEAST 1.10 [19]. For the continuous analysis, we used a Cauchy distribution to model the among-branch heterogeneity in diffusion velocity [9], and for the discrete analysis, we used the Bayesian stochastic search variable selection (BSSVS) approach to identify the best-supported lineage transitions events between NYC boroughs [8]. Additionally, BSSVS enables to determine which transition rates are zero depending on the signal in the data, and hence to estimate the number of transitions that appropriately explains the viral diffusion process. We again employed a Metropolis-Hastings algorithm for MCMC chains that were run for sufficiently long (from $25x10^7$ to 10^9 iterations for the continuous analyses and from $12x10^7$ to $6x10^8$ iterations for the discrete analyses) to reach adequate ESS values as estimated by the program Tracer 1.7 [27], sampling trees every 5x104 iterations and discarding 10% of sampled trees as burn-in. For both kinds of phylogeographic analyses, we modelled the substitution process according to a GTR+Γ parametrization [23] and specified a flexible skygrid model as the tree prior [29].*"
